# The Disease Modification Conundrum in Parkinson’s Disease: Failures and Hopes

**DOI:** 10.3389/fnagi.2022.810860

**Published:** 2022-02-28

**Authors:** Zoltan Mari, Tiago A. Mestre

**Affiliations:** ^1^Parkinson’s and Movement Disorders Program, Cleveland Clinic Lou Ruvo Center for Brain Health, Las Vegas, NV, United States; ^2^Division of Neurology, Department of Medicine, Parkinson’s Disease and Movement Disorders Center, The Ottawa Hospital Research Institute, University of Ottawa, Ottawa, ON, Canada

**Keywords:** Parkinson’s disease, neuroprotection, biomarkers, animal modeling, synucleinopathy

## Abstract

In the last half-century, Parkinson’s disease (PD) has played a historical role in demonstrating our ability to translate preclinical scientific advances in pathology and pharmacology into highly effective clinical therapies. Yet, as highly efficacious symptomatic treatments were successfully developed and adopted in clinical practice, PD remained a progressive disease without a cure. In contrast with the success story of symptomatic therapies, the lack of translation of disease-modifying interventions effective in preclinical models into clinical success has continued to accumulate failures in the past two decades. The ability to stop, prevent or mitigate progression in PD remains the “holy grail” in PD science at the present time. The large number of high-quality disease modification clinical trials in the past two decades with its lessons learned, as well as the growing knowledge of PD molecular pathology should enable us to have a deeper understanding of the reasons for past failures and what we need to do to reach better outcomes. Periodic reviews and mini-reviews of the unsolved disease modification conundrum in PD are important, considering how this field is rapidly evolving along with our views and understanding of the possible explanations.

## Introduction

PD and movement disorder specialist clinicians frequently encounter patients inquiring about a “cure” for PD, or at least when it will be possible to slow down the progression of their disease. Almost as often as we are asked about a “cure” for PD, patients share popular media news, reporting curative treatments being imminent or on the verge of discovery. Such experiences in movement disorders clinics around the globe are a reminder of the critical unmet need for effective disease modification in PD, but also for accurate and effective messaging to the PD community. While there are effective, well-established symptomatic PD treatments (Armstrong and Okun, 2020), the present enormous societal burden associated with PD (GBD 2016 Parkinson’s Disease Collaborators, 2018) is only expected to grow in an aging population (Dorsey et al., 2018), if no disease-modifying therapies in PD are available in clinical practice.

What are the missing pieces to the puzzle of successful disease modification in PD? To attempt to understand the conundrum, we start by reflecting on the history of PD. The first clinical description by James Parkinson was based on a few people, some of them random pedestrians fortuitously observed on the street (Parkinson, 1922). With the introduction of levodopa/carbidopa, symptomatic treatments (Goetz, 2011) came about to improve the diagnostic signs and symptoms of parkinsonism, in what became a true revolution to improve the lives of people living with Parkinson’s (PwP). As more treatments for core motor features of parkinsonism emerged and were further enhanced over the subsequent decades, none could be associated with slowing the relentless progression of PD.

Yet, hope emerged a couple of decades ago due to our growing understanding of PD pathophysiology and subsequent development of PD models. Early animal models relied on toxin-based interventions such as 1-methyl-4-phenyl-1,2,3,6-tetrahydropyridine (MPTP), which selectively destroyed neural structures (Langston, 2017), including and chiefly the nigrostriatal system, known to be core to PD pathology. Even though there were concerns raised about the true value of the MPTP model early on (Mari and Bodis-Wollner, 1997), it became the gold standard testing ground for therapeutic developments, targeting (A) symptomatic and (B) disease modification treatments alike. At the time, those two conceptually different therapeutic goals were not necessarily seen as distinctly separate from each other from the preclinical modeling perspective as they are today. With time, the limitations of animal modeling of neurodegenerative diseases have been recognized. For a recent review (see Dawson et al., 2018).

In fact, since the discovery of the MPTP model, the lack of successful translation from preclinical positive results to efficacy demonstrated in PD human trials has undermined the initial optimism about animal modeling. The “Valley of death” in PD disease modification populated by innumerous failures, such as the various NET-PD trials (Tilley and Galpern, 2007), the ADAGIO trial (Rascol et al., 2016), the more recent α-Synuclein immunotherapy trials (ClinicalTrials.gov, 2021) and Ven-glustat trial (Peterschmitt et al., 2021a,b), are testimony to the gaps in the planning and design of disease modification trials not properly recognized and addressed so far. There has been increasing interest and attention in the PD scientific community to this conundrum (Espay, 2021; Lungu et al., 2021) and this invited Mini Review aims to further add to the discussion.

## From Preclinical Success to Clinical Disappointment: Possible Reasons Behind Failed Therapeutic Translation and the Hope to Correct Them

In the following subsections, we review some possible contributors to the lack of translational success from preclinical to clinical therapeutic development for disease modification in PD, along with a brief (“mini”) assessment on the hopes of correcting or mitigating each of these obstacles. When applicable, we list specific reasons for hope in each of these subsections, with the most relevant specific points numbered (1) (2) (3) accordingly. While we intend these assessments to help shape a framework for therapeutic development in PD, they are not expected to fully address these complex challenges (Ransohoff, 2018).

### Timing Woes

In preclinical studies, researchers are in full control of *timing*, both in terms of the administration of a putative disease-modifying (or neuroprotective) intervention and the inception of a PD-like condition. In other words, preclinical studies can examine the effect of an intervention with arbitrary (optimized) timing relative to the onset of the pathology induced experimentally, which is not possible in real-world PD therapeutic clinical development. Administering a disease-modifying intervention at the earliest possible time in the natural history of a neurodegenerative disease is likely more effective and thus desirable, as it opens a window of opportunity for an intervention to effectively interfere *earlier* in the neurodegenerative process, before the “horses are out of the barn.” A path forward in PD is the ability to recognize earlier pre-symptomatic, pre-clinical, and prodromal states of PD and to accurately define the risk of phenoconversion over time. Regarding the risk of phenoconversion over time, it is essential to be able to detect the earliest and more subtle changes relevant to disease inception, but also to use validated biomarkers of ongoing pathology and disease stage/progression. The latter is perhaps the greatest challenge for already symptomatic disease stages, as time to phenoconversion may already be considered as an established efficacy outcome in disease modification clinical trials involving prodromal/pre-symptomatic stages.

Estimating and monitoring disease risk and expected time to phenoconversion would be greatly helpful in preclinical management and advice. For example, a patient with a known mutation in one of the PD risk genes may have a certain lifetime risk of PD at an earlier point in life, which may change later in life (for example due to certain environmental exposures or epigenetic effects). Hopefully easily, inexpensively, and passively monitorable parameters or functions (for example keyboard use, speech, change in autonomic functions, motor speed, habits, sleep, etc.) could raise alerts pointing to a possibly changing risk of impeding phenoconversion. At that point, individual patient’s risk could be further evaluated by high specificity and sensitivity testing (such as dopamine nuclear imaging). Once their risk of phenoconversion is established and reaches a certain threshold, the risk and benefit of any disease modifying intervention may be determined taking that into account. This may be particularly important if future, putative disease modifying interventions carry substantial risk of side effects or irreversible complications.

#### Mini Assessment

It is not expected that clinicians and human clinical trials will ever be in full control of the timing of administration of candidate disease modifying interventions relative to disease inception. However, there still is hope, when it comes to timing woes.

(1) Efforts to target *de novo* patients (and especially subjects at a prodromal stage) can move the timing of a disease modification intervention to a point closer to the presumed inception.
(2) A more ambitious hope is to target those at-risk for PD using a population-based risk-monitoring approach, possibly identifying at-risk individuals earlier in the natural history of the disease. To that end, likely a combination of a validated biomarker is needed with rigorous diagnostic criteria for at-risk PD (Mantri et al., 2019). The ubiquitous nature and technological improvements of consumer-grade monitoring devices could potentially act as part of such population-based screening for impending phenoconversion by alerting individuals of concerning changes in their level of daily activities (motor or non-motor) and functioning, related to pre-symptomatic PD.
(3) Finally, it is also possible that general preventative measures can be safely applied to the general population (such as exercise, removal of toxic contamination from food and drinking water) which, in turn, may impact the risk of incident PD.

### No Perfect Model of Parkinson’s Disease: The Unnatural Nature of “Pure and Simple” Preclinical Designs

The heterogeneity of sporadic PD remains an active field of research to accurately understand pathophysiology and the impact of independent and simultaneous pathologies, with the intention to inform prognosis, and shape therapeutic development for target-/group-, and individual specific disease-modifying therapies (Mestre et al., 2021). Heterogeneity among individual patients is significant to the point that PD may be more appropriately called a syndrome with multiple causes (Devos et al., 2021).

In preclinical experiments, the design is traditionally driven to simplify and dissect a specific pathological pathway. To accomplish this preclinical goal, “noise” needs to be reduced or eliminated. As part of such deliberate effort, in preclinical models, biological heterogeneity, co-morbidities, and inter-individual variability is attempted to be minimized or eliminated. But this simplification to create “pure” models may then undermine how preclinical models accurately represent their respective human condition. For example, cross-seeding in multiple proteinopathies is a recognized phenomenon in parkinsonian disorders (Williams et al., 2020) and the interplay with other pathologies outside of proteinopathies is also known (Motyl et al., 2021).

In the clinical arena, despite efforts to adopt restrictive eligibility criteria in clinical trials, study participants have intrinsically greater heterogeneity compared with preclinical (animal) models, which typically use nearly identical individuals within the animal cohort. In human studies, beyond the heterogeneity of PD itself as established clinically, there is also the intra-cohort heterogeneity with respect to factors outside of PD itself, including the individuals’ age, genome, their environmental exposures, and comorbidities. Usually these areas of heterogeneity among study subjects are not fully accounted for in human clinical trials, even if most do exclude independent neurological comorbidities. The way these various sources of heterogeneity may affect how patients respond to a disease modifying intervention, even when they otherwise possess a common pathological target relevant to the tested intervention, is still largely unknown. Such consideration is generally excluded from creating preclinical models. Consequently, at the present time, a preclinical model of PD is likely to fail as it is not an accurate representation of a very important aspect of sporadic PD, which is heterogeneity of the sample.

In face of the disconnect between the human (heterogeneous) PD and its (homogeneous) preclinical models, an approach that could gap this bridge in drug development may be the ability to select study participants based on the proposed mechanism of action of the experimental intervention being tested, what constitutes an enrichment strategy. This approach would diminish the heterogeneity among individuals of a study cohort in one important way. A biomarker-driven phenotyping (Espay et al., 2017, 2020) may inform this approach. The rule in the past has been that disease modification clinical trials almost exclusively used enrollment criteria based on clinical diagnostic criteria for PD, thus the enrichment strategy promoted by a few authors has been uncommon.

Nevertheless, some recent efforts indicate that the field may be moving forward from clinical diagnostic criteria-based enrollment. One example is the SURE-PD3 trial, where the study design purposely used an enrichment strategy (serum urate below the population median concentration), based on the expected mechanism of action of inosine, the study intervention (Parkinson Study Group et al., 2021). Although, this particular study failed to meet its primary endpoint, we believe it to be an important example of how a biomarker-defined specific sub-population of PD, beyond just meeting clinical diagnostic criteria, can be feasibly employed in a large multicenter clinical trial. As discussed elsewhere in this mini-review, there of course may be a number of potential other reasons for failure, besides the reliance on clinical diagnostic criteria only in disease modifying trials. One example of enrichment of the study population could be the use of PET scanning to identify PD patients with evidence of microglial activation for interventions targeting microglia (Yun et al., 2018; Muzio et al., 2021).

Another example of selective inclusion criteria beyond just meeting clinical diagnostic criteria is represented by a trial of azathioprine, an immunosuppressant medication, to investigate whether suppressing the peripheral immune system can be disease-modifying in PD. In this trial, a prognostic risk score (Greenland et al., 2020) was adopted and those at higher risk of progression were included.

A caveat for restricting PD enrollment to patients with a unique molecular abnormality carries financial and logistical challenges, which can make such an approach less attractive (Lungu et al., 2021). Also, while we strongly believe that anchoring enrollment for disease modifying trials solely on clinical diagnostic criteria is suboptimal, merely correcting this issue alone may not automatically guarantee success, for the possibility of several other errors in trial design. To highlight that cautionary note, the selection of PD patients carrying specific GBA mutations did not save the Ven-glustat trial from failure, with the unexpected finding that those allocated to active treatment and carriers of a less severe GBA mutation had a greater progression on efficacy outcomes (Peterschmitt et al., 2021a,b), again bringing attention to the complexity and multifactorial nature of the problem.

#### Mini Assessment

To address the consistently failing preclinical-to-clinical translation in PD therapeutic development, it is paramount that a major characteristic of human populations in clinical trials, i.e., natural heterogeneity among individuals of the sample, is represented in preclinical models, especially if the target population is sporadic PD, for a better understanding of the impact of a novel experimental therapy from a biological and outcome perspective. At the same time, and along the lines of a more rational and solid drug development, disease modification trials should not settle at using only clinical diagnostic criteria for study eligibility, without an “enrichment strategy,” e.g., incorporating biomarker-driven inclusion criteria, linked to the putative mechanism of action of the experimental therapy being developed.

(1) Preclinical models should replicate human disease in more than one way. While it is necessary that the individuals of a preclinical experimental cohort share the targeted specific abnormality, heterogeneity in other aspects among individuals of the sample (their age, genetic background, presence/absence of unrelated other pathologies/comorbidities) is desirable, for better representation of a human clinical trial population. This premise is different from current standards, complex, and challenging—yet theoretically possible. For example, injection of α-synuclein fibril seeds combined with adeno-associated virus (AAV)-mediated overexpression of human α-synuclein (Thakur et al., 2017) may be done in an otherwise heterogeneous group of rats. Also, recent developments in mRNA technology could also add tools to create specific changes in protein expression (Kallen and Thess, 2014) in otherwise heterogeneous group of experimental animals. With such approach, it may be possible for investigators to create the same molecular pathological process in an otherwise heterogeneous cohort. The hope is that preclinical efficacy of an intervention may translate better from such models to human trials.
(2) Generic and broad inclusion of clinically defined PD populations for disease modification studies have been motivated by feasibility and financial considerations, while also a necessary compromise due to lack of validated biomarkers in most circumstances. However, the use of an enrichment strategy with more restrictive enrollment criteria, narrowing the study population on the basis of well-defined pharmacodynamic biomarkers of the studied therapeutic target, could offer an attractive starting point for success in disease modification in PD. Similar suggestions have been made recently by others (Espay et al., 2020; Devos et al., 2021). The enormous investment in disease modification clinical trials over the past couple of decades should convince stakeholders that the strategy of “just keep doing the same” will likely only lead to further waste of time and resources, whereas the concept of a well-informed “less is more” rationale proposed here could not only be scientifically sound, but financially pertinent, as it may yield a new opportunity of turning failure to success.
(3) To achieve this ambitious but necessary shift in strategy and vision, stronger collaborations between preclinical and clinical scientist are necessary. “Bench-to-bedside-and-back-again” frameworks may lead to innovation that could address this disconnect.

While seemingly contradictory on the issue of heterogeneity in study cohorts, points (1) and (2) are in fact complementary. To best imagine this, we propose that current standards of animal modeling and clinical testing of disease modification interventions in PD are two ends of a spectrum with regards to sample heterogeneity among individuals within study cohorts. Preclinical experiments make a painstaking effort to eliminate any and all such heterogeneity, whereas human clinical trials employing phenomenology-based clinical diagnostic criteria to select their study population make no actual effort to homogenize their sample in a way relevant to the tested intervention. While the latter point has been discussed elsewhere (Devos et al., 2021; Espay, 2021; Mestre et al., 2021), the purpose of animal modeling is to achieve a more accurate representation of a human disease that better supports testing in humans in a continuum where prior studies inform future studies and reduce risk of failures and yield for success. To improve PD preclinical models, better representation of the human sample’s heterogeneity (in age, comorbidities, past environmental exposures, etc.) is necessary.

### Targets: The Age of Discovery

The discovery of the nigrostriatal dopaminergic system and the possibility to replenish the deficient neurochemical state of dopamine observed in PD (Carlsson et al., 1957; Hornykiewicz, 1966; Cotzias et al., 1969) led to a prolific therapeutic development. The Nobel prize in the year 2000 was awarded to Arvid Carlsson (1923–2018) and Lees et al. (2015) for the discovery of dopamine and its role in human movement and it is a testimony to the impact of these scientific breakthroughs. It is not an hyperbole that this discovery created a revolutionary momentum in the development of neurological treatments not previously imagined. In contrast, the series of failures in disease modification trials cast a shadow on the feasibility of neuroprotective treatment targets thus far identified and the ability of candidate treatments aimed at those targets to slow down PD progression (Lungu et al., 2021). Therefore, despite all the earlier breakthroughs relative to dopamine that became major milestones in human neuroscience, we are living in a new age in need of discovering new targets in PD with a renewed promise of disease modification. Examples include the development of therapeutics targeting specific genetic forms of PD such as GBA-related PD (Peterschmitt et al., 2021a,b) and LRRK2-associated PD (Schneider and Alcalay, 2020) with ongoing clinical trials, as well as the identification of new molecular fingerprints and other pathophysiological mechanisms in PD neurodegeneration that may be targeted in the future for development of novel therapies (Zeng et al., 2018). This growing knowledge may allow in the future to define the disease subtypes based on specific disease mechanisms at the molecular level; targets potentially associated with the potential for a disease modification intervention, even prior to the emergence of a motor phenotype and independent of a clinical diagnosis.

#### Mini Assessment

To improve better disease modifying clinical trials in PD and our chance for success, new treatment targets are need.

(1) Developing a new classification system of PD etiologies will be needed considering cases with a well-defined cause to broader categories of multifactorial and idiopathic etiology.
(2) It is also critical to revise clinical trial designs to place great emphasis on a robust connection between therapeutic target and study population definition, and (in select cases of proof-of-concept trials) a linked endpoint. We will further discuss endpoints in the next section.
(3) A “cocktail” of multi-target therapeutic intervention may prove to be a more successful intervention in the future for disease modification. It is unlikely, even in case of study population selected based on a single cause, such as gene-defined forms of PD, that a single target will be sufficient for successful disease modification.

### The Importance of the “Right” Endpoints: The Era of Biomarkers

How do we objectively measure progression in PD? A commonly cited reason for failure of PD disease modification trials is the lack of perfect progression endpoints; that the “gold standard,” the Movement Disorder Society’s Unified Parkinson’s Disease Rating Scale (MDS-UPDRS), should be viewed more as a “bronze standard.” A known floor effect, intra- and inter-rater reliability issues, and great susceptibility to symptomatic treatment related variability (Teshuva et al., 2019; Trifonova et al., 2020) make the MDS-UPDRS suboptimal as a primary outcome measure in trials of disease modification. Consequently, there have been calls to use a more complex multi-domain characterization of the PD clinical syndrome One proposed solution is the so-called “digital phenotyping” of PD (Bhidayasiri and Mari, 2020), with the necessary technology and resources becoming available, it is becoming a reality to use more objective digital measures capturing a variety of measures in a multitude of motor and non-motor domains (Dorsey et al., 2020). Research on progression biomarkers remains one of the most relevant field in the area of disease modification research in PD (Marek et al., 2011; Espay et al., 2020). A leading challenge affecting the adoption of reliable biomarkers, including digital, clinical, imaging, biological/biofluid, neuropsychological, and others, remains centered on their proper validation. The complexity of this challenge, further complicated by the influence of commercial developments and interests, represents no smaller barrier than any of the others outlined in previous sections in this mini-review. However, there are increasingly credible efforts to solve this difficult riddle (Espay et al., 2019; Adams et al., 2020; Schneider et al., 2021). The Parkinson Progression Marker Initiative (PPMI) is one of those projects. PPMI is the largest longitudinal study to pursue imaging and biofluid markers of progression in PD (Marek et al., 2011) that aims to provide a comprehensive PD phenotype including not only non-treated PD with a recent clinical diagnosis, but also for subjects deemed to be at a prodromal stage. While so far PPMI has not produced revolutionary breakthroughs, its design, scope, and methods are constantly adapting to new data and are setting new specific goals, having produced significant advances in our understanding of PD pathophysiology, natural history, and ways to more precisely measure disease progression (Rahmim et al., 2017).

#### Mini Assessment

Along with the other key contributors to past failures of disease modification trials in PD, solving the problem of best endpoints is equally critical. Several ideas come into consideration.

(1) Rather than validating candidate progression biomarkers against existing standards, they may need to be validated against disease-relevant and even disease subtype-, stage-, study-, target-, or individual-specific outcomes, as discussed in a recent review (Bhidayasiri and Mari, 2020).
(2) Deep phenotyping or digital phenotyping (Bhidayasiri and Mari, 2020; Dorsey et al., 2020) would need to sensibly combine a variety of biomarkers and measures.
(3) A scientist and science-driven approach that appropriately regulates industry participation will be required for success (Espay et al., 2019; Lungu et al., 2021). The explosion of consumer electronic technology has led to the burgeoning offerings of consumer fitness and health monitoring products that overlap and encroach the zone of healthcare grade devices, often developed by the same companies as consumer grade monitoring technology. The financial conflicts of interests apply in this area of research and development similarly to other areas, but regulatory response to the rapidly changing field of study simply could not keep up the pace, adding another layer of complexity.

## Discussion

The conundrum we are faced with a lost in translation of promising preclinical results into human clinical trial success for PD disease modification qualifies as a crisis. In PD, we are now searching ways to identify the opportunities in this crisis. As outlined at the end of each section above, we do have an opportunity as well as a mandate to change directions, introduce new concepts and experimental protocols, *both* in the preclinical and clinical research realms. Learning from the lessons of over more than two decades of failed disease modification clinical trials in PD, we believe that increased collaboration and coordination across basic and clinical scientists, further increased funding and support from public, foundation, and private industry sources will need to come together to ignite a paradigm shift. It is also necessary that the scientific agenda in PD be primarily academically driven, while industry influence is properly balanced by a scientifically rigorous agenda. For example, in North America, an academically based not-for-profit research network of credentialed movement disorder specialists and clinical trial experts, such as the PSG and the Michael J. Fox Foundation, can be an ally and advisor to industry sponsors in the development and advise on and oversee the conduction of modern clinical trial designs. Various platforms for scientists and projects, including special interest groups, workshops, and themed symposia within existing large international conferences or new platforms bringing together scientists from all sides can be an effective way of learning from each other and promote broader collaborations.

With an enormous public health need for effective treatments to slow, and ideally prevent the progression of PD, the PD research community is under tremendous pressure to solve this conundrum. We hope that an improved understanding of the likely contributors to past failures can pave the way to success with the creation of animal models that more accurately represent the human disease, the design better clinical trials, with targeted patient selection, and more sensitive and specific biomarkers as endpoints.

## Author Contributions

ZM developed first draft. Both authors contributed to final draft.

## Conflict of Interest

The authors were the Chair (TM) and Co-Chair (ZM) of the PSG’s “Motor Features Working Group” and in that capacity advise a variety of sponsors on clinical trial design related matters. ZM has received consulting fees from Global Kinetics Corporation, served as Medical Chief Officer for Neuraly, Inc., and holds shares of nQ Medical Inc., GB Sciences, and D&D Pharmatech, Inc. TM reports speaker honorarium from Abbvie, and International Parkinson and Movement Disorder Society; consultancies from CHDI Foundation/Management, Sunovion, Valeo Pharma, Roche, nQ Medical and Merz; advisory board from Abbvie; Biogen, Sunovion, Medtronic, and research funding from EU Joint Programme—Neurodegenerative Disease Research, uOBMRI, Roche, Ontario Research Fund, CIHR, MJFF, Parkinson Canada, PDF/PSG, LesLois Foundation, PSI Foundation, Parkinson Research Consortium and Brain Canada.

## Publisher’s Note

All claims expressed in this article are solely those of the authors and do not necessarily represent those of their affiliated organizations, or those of the publisher, the editors and the reviewers. Any product that may be evaluated in this article, or claim that may be made by its manufacturer, is not guaranteed or endorsed by the publisher.
